# Comparison of Efficiency of BG-Sentinel Traps Baited with Mice, Mouse-Litter, and CO_2_ Lures for Field Sampling of Male and Female *Aedes albopictus* Mosquitoes

**DOI:** 10.3390/insects8030095

**Published:** 2017-09-01

**Authors:** Gilbert Le Goff, David Damiens, Abdoul-Hamid Ruttee, Laurent Payet, Cyrille Lebon, Jean-Sébastien Dehecq, Martin Geier, Louis-Clément Gouagna

**Affiliations:** 1 MIVEGEC, IRD (Institut de Recherche pour le Développement), CNRS, Univ. Montpellier, F-34394 Montpellier, France; gilbert.legoff@ird.fr (G.L.G.); louis-clement.gouagna@ird.fr (L.-C.G.); 2 IRD Réunion/GIP CYROI (Recherche Santé Bio-innovation), 97490 Sainte Clotilde, Reunion Island, France; cyrille.lebon@ird.fr; 3 Service de lutte anti vectorielle, Agence Régionale de Santé-Océan Indien (ARS-OI), 97743 Saint-Denis, Reunion Island, France; Abdoul-Hamid.RUTTEE@ars.sante.fr (A.-H.R.); Laurent.Payet@ars.sante.fr (L.P.); Jean-Sebastien.DEHECQ@ars.sante.fr (J.-S.D.); 4 BiogentsAG, Weissenburgstrasse 22, D-93055 Regensburg, Germany; martin.geier@biogents.com

**Keywords:** trapping, male mosquito, sterile insect technique (SIT)

## Abstract

Determining the abundance and distribution of male mosquitoes in the wild and establishing species seasonality in candidate pilot sites is of particular interest with respect to the use of the sterile-male technique. With the knowledge that using mice as bait in BG-Sentinel traps effectively enhances *Aedes albopictus* male and female trapping success, the present study was designed to determine whether attractants derived from mouse odour blend could be a viable substitute for live mice to lure *Ae. albopictus* mosquitoes into traps. The effects of baiting BG-Sentinel traps with mice, carbon dioxide (CO_2_), and attractants derived from litter mouse odours (mouse litter (ML)) and a mouse odour blend (MOB) on the efficiency of trapping *Ae. albopictus* males and females were tested using a Latin square design. The BG-Sentinel trap baited with CO_2_ + ML caught a significantly larger number of mosquitoes compared to traps baited with mice only. The BG-Sentinel traps containing only CO_2_ or CO_2_ + MOB, however, did not catch significantly more mosquitoes compared to the other traps. The proportions of males caught in the BG-Sentinel traps did not differ significantly between the respective attractants. The results from this study confirm that CO_2_ bait is efficient to provide a reliable estimation method for *Ae. albopictus* adult male abundance in the wild, and suggest that mouse litter baits in combination with CO_2_ could be used to enhance *Aedes* trapping success in BG-Sentinel traps.

## 1. Introduction

The sterile insect technique (SIT) is a biological control method for use against insect pests based on the release of large numbers of sterile males into the wild population. These will compete with the wild males to mate with females in the field and thereby reduce the fertility of the target population [1,2]. The success of SIT relies mainly on a convenient temporal and spatial release strategy that includes a sufficient number of sterile males released inappropriate locations at an appropriate frequency. One essential consideration when designing an optimal release strategy is a realistic estimation of the wild population density and its temporal dynamics [3]. The actual number of a given species per unit area may be estimated by mark-release-recapture (MRR) experiments, which consist of the release of mass produced insects (usually marked with fluorescent dyes) and extrapolating the population density from daily recapture data using appropriate analysis techniques [4,5,6]. The ability to conveniently utilise this MRR technique to estimate the population size of male *Aedes albopictus* (Skuse) relies heavily on the availability of efficient and effective male trapping systems.

Recent investigations into the ecology of *Ae. albopictus*, the main vector implicated in the recent devastating chikungunya outbreak in La Reunion Island, indicated that this species may be a good candidate for control through SIT [7,8,9,10]. To this end, efforts have been made to determine their presence and densities, as well as to establish species seasonality in candidate pilot sites [11], so as to best ascertain when to initiate sterile male release and to be able to measure the impact of such interventions. Although previous lines of evidence elsewhere have suggested that the classic BG-Sentinel (BGS) [12] traps are efficient in collecting *Ae. albopictus* [13,14,15,16], the capture rates of *Ae. albopictus* using BGS traps with a commercial synthetic BG-lure on La Reunion Island were surprisingly relatively low, and mainly dominated by females [17,18]. This decreased ability to accurately sample *Ae. albopictus* males on the Island posed an obstacle toward SIT implementation until it was overcome by baiting the traps with mice (*Mus musculus*) [19,20,21]; where baiting with three mice, adult *Ae*. *albopictus* collections increased 10-fold and the proportion of males collected increased by 20%–25% compared to the traps baited with lure alone. These results primarily reflect the attractiveness of female mosquitoes toward CO_2_ and odour emanating from the mice. The increase of female attraction may have led to a concurrent male attraction increase, since males are known to forage near the host to intercept females.

Although generally effective, the use of live mice-baited BGS traps in an intensive monitoring program could be limited due to logistical constraints and protracted costs associated with the breeding of mice. To avoid the need to rear mice, while retaining the attractiveness, it was postulated that asynthetic analogue of mouse-borne odour blend could be used in their stead for large-scale sampling. Expanding on an earlier investigation [21], the present study was designed to determine whether mouse litter (absorbent wood material into which mice can urinate or defecate), or an odour blend from the same, could be substitutes for live mice to enhance *Ae. albopictus* collections in BGS traps. These were tested in combination with carbon dioxide under field conditions against BGS traps baited with live mice.

## 2. Materials and Methods

### 2.1. Study Area

The field experiment was carried out from 7–24 March 2016 in Duparc, an urban district in the north east of the capital Saint-Denis, La Réunion Island (55°32′ E, 21°08′ S). It is a 22-ha urban zone that lies 50–80 m above sea level, with about 373 premises covering 22% of the surface area. It is isolated by an expressway linking Saint-Denis to Saint-Benoit in the north, by La Mare Ravine in the east, and by sugar cane fields to the east, west, and south. This area experiences abundant populations of *Ae. albopictus* that fluctuate seasonally, and was recently chosen as one of the candidate sites for a pilot demonstration of *Ae. albopictus* sterile male releases.

### 2.2. Trap Descriptions

In this experiment, the use of lures derived from mice in BGS traps was tested and compared to the trapping efficiency of live mice baited-BGS traps used in previous study [21]. Four different conventional BGS traps were tested, each with one of the following attractants: live mice, CO_2_ alone, mouse litter (ML) + CO_2_, and mouse odour blend (MOB) (developed by Biogents) + CO_2_. Quantities of attractants were not optimised in this proof-of-concept study, and remained for further study pending the outcome.

In BGS traps with live mice, three adult white mice (*Mus musculus*) were placed in a rearing polycarbonate cage (265 × 205 × 140 mm) placed at the bottom of classic BGS trap as described in Reference [21].

For the carbon dioxide-baited mosquito traps, a half-gallon plastic insulated container was filled every morning with approximately 1.7–2.0 kg dry ice and placed directly inside the BG sentinel between 9:00 and 10:00 p.m. The natural sublimation of dry ice generated CO_2_, which was released from a 5-mm hole made on the side of the container, and dispersed by the movement of the BG trap air-flow.

The mouse litter (ML) was obtained by lining a small cage with 500 g of chipped wood (pine) to absorb the urine and faeces of the four white laboratory mice which were kept in the cage. After five days, the mouse litter was removed and mixed to homogenize the urine and faeces throughout the litter. About 30 g of the litter was packed in a Tyvek sachet and stored under ambient conditions inside a hermetically sealed box (20 × 10 × 7 cm). During deployment, the Tyvek sachet was opened when the trap was installed and the material placed in a small opened plastic tube (100 mL) which was inserted in the small black mesh pocket located on the outside of the suction tube of the BG sentinel trap(the pocket initially dedicated to the position of the BG lure).

The mouse odour blend (MOB) is the odour blend extracted from 500 g of chipped wood (pine) previously used as litter material for four caged mice. After five consecutive days, the odour of mouse litter was extracted with 1 L ethanol, which was then concentrated to 50 mL at room temperature. This extract was subsequently absorbed in polypropylene beads and dried. Polypropylene beads have tiny pores that homogenously dispense odour over time. About 15 g of the scent product was placed in a Tyvek sachet and kept under ambient conditions as indicated above until use. For the experiment, the Tyvek sachet was opened when the trap was installed and the material was placed in a small plastic tube (100 mL) which was inserted in the small black mesh pocket located on the outside of the suction tube of the BG sentinel trap.

### 2.3. Mosquito Sampling

A Latin square design was used, as previously described [21], with four fixed trap positions determined in a residential area in Duparc, Sainte-Marie. Traps were placed on the ground in shaded locations that were separated from each other by a minimum of 50 m and a maximum of 100 m. Three replicates were performed between 7–10 and 14–17 March, as well as 21–24 May 2016, and each experimental trial consisted of a complete trapping cycle of 4 × 24-h trapping periods. During this time, the trap setup was rotated every 24 h so that, at the end of a four-day sampling, each attractant had been tested in every position.

The power source for all BGS traps were 12 V batteries that were changed every day with fully charged batteries. The group of mice used as bait were left in the field for the first two consecutive days and were replaced by new mice for the last two days. Traps were activated every day between 09:00 and 10:30, and insect capture bags were collected the following day at exactly the same time. The field-collected samples were brought to the laboratory, where mosquitoes were individually identified using morphological characteristics. The number of *Ae*. *albopictus* adults and the male ratio (defined as the number of males caught divided by the total number of *Ae*. *albopictus* adults caught) were recorded for each collection.

### 2.4. Statistics

A G-test [22] was used to compare the observed counts of each category (type of baits or the week of collection) with the expected counts. The expected count was calculated as the theoretical expectation if the same proportion of mosquitoes was caught in each trap, whatever the type of bait or week of collection (such as a 1:1:1:1 ratio for the type of trap or a 1:1:1 ratio for the three weeks of collection). For the post-hoc test, the categories which were significantly different from their null hypothesis were determined by testing each category vs. the sum of all categories, with the Bonferroni correction.

The effect of the four types of attractant was tested while controlling for the variability of the four different positions (in this case the Latin square number) and the four different trapping periods. As the Latin square design was replicated three times in the conditions described, the same row (Date) and column (Position) levels were kept, giving three identical squares for the three replicates that could be analysed through a General Linear Models (GLM) test in Minitab 16 [23]. The GLM procedure was performed using two response variables: the total number of *Ae*. *albopictus* adults collected in each trap, and the male ratio within the caught samples; while the fixed independent variables were the trap position and date of sampling, and the covariate was the attractant (three mice, CO_2_ alone, and CO_2_ in combination with ML and MOB). Multiple comparison procedures (Tukey’s HSD tests) were also performed to test significant differences (with a significance level of 0.05), in the number of caught mosquitoes among different traps. All analyses were run in Minitab statistical package.

### 2.5. Ethics Statement

This study was conducted in the context of an ongoing long-term SIT project initiated in 2009, and benefited from mosquito sampling protocols involving one or more experimental procedures with animal models. No prior ethical clearance was required. With regard to the European Union (EU) Directive “2010/63/EU of 22 September 2010 on animal care and use in experimental procedures, a directive transposed into French law in 2013 (Decree No. 2013-118 of 1 February 2013) regarding the ethics review and clearance of research involving the use of animals, programs that began before 2013 are not subject to validation by the Ethics Committee. The rearing has been done by a trained staff with the necessary skills to manipulate all mice involved in this study in accordance with guidelines for the protection of animals used for experimental purposes. The use of laboratory mice in rearing cages, as was done in the present study, ensured the welfare of the animals and avoided unnecessary stress.

## 3. Results

### 3.1. General Observations

A total of 2634 adult mosquitoes, belonging to four culicidian species, were collected over the course of the study. *Aedes albopictus* (2276 adults: 1317 females + 959 males) and *Culex quinquefasciatus* (Say) (355 adults: 260 females + 95 males) were the dominant species regularly collected in all traps, while the other species caught were two specimens of *Lutzia tigripes* (Grandpré and Charmoy), and one of *Culex neavei* (Theobald). In total, 224 (10%), 542 (24%), 879 (38%), and 631 (28%) *Ae*. *albopictus* were captured in the three mice, CO_2_, CO_2_ + ML, and CO_2_ + MOB baited BGS traps, respectively, in significantly different proportions (G-test, *G* = 424.75, *df* = 3, *p* < 0.001). For *Cx*. *quinquefasciatus*, no significant difference between traps was observed (G-test, *G* = 2.71, *df* = 3, *p* = 0.44) with 87 (25%), 94 (26%), 77 (22%), and 97 (27%) adults were collected in the three mice, CO_2_, CO_2_ + ML and CO_2_ + MOB baited BGS traps, respectively. The number of adults captured during the three replicates were significantly different for *Ae*. *albopictus* (613, 542, and 1121 (G-test, *G* = 249.38, *df* = 2, *p* < 0.001)) and for *Cx*. *Quinquefasciatus* (117, 85, and 153) for week 1, 2, and 3, respectively (G-test, *G* = 19.72, *df* = 2, *p* < 0.001).

### 3.2. Efficiency of Ae. albopictus Trapping According to the Attractants

**Figure 1:**
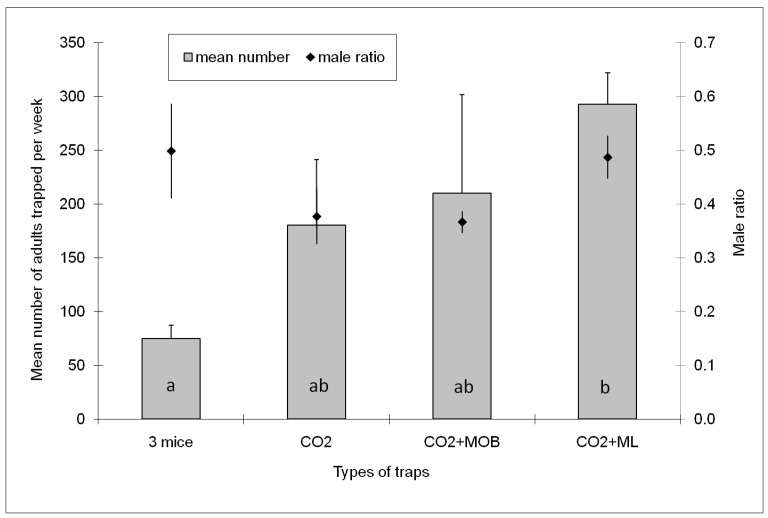
Mean number of *Ae. albopictus* adults (males and females) (bars) and mean male ratio (defined as the number of males caught divided by the total number of adults caught) (line) per week by different types of baits. Columns with the same letter indicate that the results are not significantly different (*p* > 0.05, Tukey’s HSD post-hoc test following a GLM procedure within each zone).

Figure 1 depicts the distribution of the average numbers of adults (males and females) and the ratio of male mosquitoes to the total number of adults caught by traps with the different types of attractants over all sampling days for three replicates. The number of adults caught varied significantly with the type of attractant present in the traps (GLM, *F*_(3, 34)_ = 5.77, *p* < 0.005). According to the post hoc tests, BGS baited with CO_2_ + ML was the only trap that caught a significant higher number of mosquitoes compared to the trap baited with three mice (Figure 1). BGS traps baited with only CO_2_ or with CO_2_ + MOB lure did not catch significantly more adult mosquitoes compared to traps baited with three mice. Finally, no significant difference in the male ratio was seen among traps using different attractants (GLM, *F*_(3, 35)_ = 1.20, *p* = 0.235, Figure 1).While the three mice-baited traps and the mice-derived odour baits attracted an overall male ratio of 0.5, only a male ratio of 0.37 was attracted with other attractants (Figure 1).

## 4. Discussion

The present study demonstrates for the first time that mouse odours such as urine or faeces act as an effective attractant for *Ae. albopictus* mosquitoes when used in combination with CO_2_. The average number of *Ae. albopictus* adults collected with BGS traps baited with three mice compared closely with the results obtained in the previous study [21], while the use of mouse litter in combination with a CO_2_ source outperformed traps baited with mice.

The attractiveness of *Ae. albopictus* females to a host involves a complex suite of mechanisms [24] including host skin odours [25], excretion odours, breath volatiles [26], heat, and moist convective currents or CO_2_ emission [27,28], all of which increased with the increasing number of mice. It has been established that these stimuli activate compound-specific receptors that, in turn, stimulate host-seeking behaviour in insects [29]. Herein, the average number of *Ae*. *albopictus* adults caught with traps baited with CO_2_ alone was comparable to that of traps baited with three mice, confirming that *Ae. albopictus* can be lured by BGS traps using carbon dioxide [30,31]. This finding is consistent with a recent report that significantly more *Ae*. *albopictus* adults were caught in CO_2_-baited traps (either in the form of dry ice or compressed gas) than in unbaited traps [17]. Although these differences were not statistically significant, the increased trap capture of *Ae. albopictus* may be attributed to the synergistic action of carbon dioxide with attractant blends from mouse litter.

The chipped wood used as litter material was undoubtedly impregnated with odours emanating either from the mice enclosed in their holding cages or from their excretions (such as urine and faeces) [32,33,34] which were mixed with the odour of the wood (pine) itself. It is more likely that mosquitoes may use the excretions odours and the kairomones, which regulate the social behaviour of mice [35,36,37], as an indicator of the presence of a bloodmeal source. These infochemicals were therefore credited as the primary cues attracting *Ae. albopictus* to baited BGS traps, the level of which is a function of the number of mice present, as shown previously [21]. A positive correlation, however, was found between different methods of extraction and the proportion of males caught in BGS traps with the increasing number of mice used as bait. It is believed that male attraction to traps was a result of their response to conspecific females rather than the detection of bait odours [38,39]. In the present study, however, the proportions of males caught did not vary according to the type of bait used, with an overall male:female ratio of 1:1 or less. While a convincing argument to explain this result has not yet been found, more study is needed, especially on which specific cues are more important for attracting *Ae. albopictus* males to baited BGS traps. Moreover, more studies are also needed to deal with the quantity of attractant molecules released (by the wood and the excretions) according to the treatment and the method of extraction. The purpose of this paper was a first trial using new attractants developed from mouse odours. Future experiments should focus on the optimisation of extraction methods to improve the attraction power of the “artificial” lure.

## 5. Conclusions

Initial observations and field reports in La Reunion Island indicated that live mice are highly attractive *Ae. albopictus* males and females, but the implementation of this approach for large-scale sampling is limited, not only due to logistical, financial, and regulatory constraints associated with mice husbandry, but also the ethical consideration ofputting live mice in traps. Realistically, therefore, synthetic substitutes for live mice bait will offer a huge advantage. Although a CO_2_ source in the form of dry ice showed great results, the present study showed for the first time that mouse litter scent combined with a CO_2_ source may offer a good alternative to live mice. Our findings could be considered as an important step in the development of novel and cost-effective synthetic versions of odour blends which can match the attractiveness of live mice or litter scent for field populations of *Ae. albopictus*. These could be produced by companies that could take on the responsibility of the rearing of the mice and the lure productions. These lures could be purchased by laboratories and institutions to enhance the effectiveness of their traps for male sampling success and enable the trapping of *Ae. albopictus* mosquitoes when used in conjunction with CO_2_ or other existing attractive components [40] in order to increase the trapping success.
